# Genetic characterization of three mitochondrial gene sequences of goat/sheep-derived *coenurus cerebralis* and *cysticercus tenuicollis* isolates in Inner Mongolia, China

**DOI:** 10.1051/parasite/2018002

**Published:** 2018-01-19

**Authors:** Yichi Zhang, Wei Zhao, Di Yang, Yuan Tian, Weizhe Zhang, Aiqin Liu

**Affiliations:** 1 Department of Parasitology, Harbin Medical University China; Harbin Medical University, Heilongjiang Provincial Key Laboratory for Infection and Immunity; Heilongjiang Key Laboratory for Pathogen Biology, Harbin, Heilongjiang 150081 PR China; 2 Department of MRI Scanning Room, the First Affiliated Hospital of Harbin Medical University, Harbin, Heilongjiang 150001 PR China

**Keywords:** *Coenurus cerebralis*, *cysticercus tenuicollis*, sheep, goats, mitochondrial gene

## Abstract

*Taenia multiceps* and *Taenia hydatigena* are widely distributed tapeworms of canids. Due to a lack of genetic information on these two parasites in China, in this study we analyzed six *coenurus cerebralis* and two *cysticercus tenuicollis* cysts from goats or sheep in Inner Mongolia, northern China by amplifying three mitochondrial genes (*cox1*, *nad4*, and *cytb*). Two haplotypes were obtained at each locus for either of the two *Taenia* cestode species, with ten nucleotide sequences being novel. The degrees of genetic variations were 1.18%, 0.61% and 0.52% for *coenurus cerebralis*, and 0.24%, 0.46% and 0.35% for *cysticercus tenuicollis* at the *cox1*, *nad4* and *cytb* loci, respectively. This is the first molecular description of animal-derived metacestodes of *T. multiceps* and *T. hydatigena* in Inner Mongolia, China. Novel nucleotide sequences might reflect endemic genetic characterization of the two cestodes. The present data are useful to explore the biological and epidemiological significance of intra-specific variations within both *Taenia* cestodes.

## Abstract (fr)

*Taenia multiceps* et *Taenia hydatigena* sont des ténias de canidés largement distribués. En raison de la quantité limitée de données génétiques sur ces deux parasites en Chine, les kystes de six *coenurus cerebralis* et deux *cysticercus tenuicollis* de chèvres ou de moutons de Mongolie intérieure, au nord de la Chine, ont été analysés en amplifiant trois gènes mitochondriaux (*cox1*, *nad4* et *cytb*). Deux haplotypes ont été obtenus à chaque locus pour l’une ou l’autre des deux espèces de *Taenia*, avec dix séquences nucléotidiques nouvelles. Les degrés de variation génétique étaient respectivement de 1,18%, 0,61% et 0,52% pour *coenurus cerebralis* et de 0,24%, 0,46% et 0,35% pour *cysticercus tenuicollis*, aux locus *cox1*, *nad4* et *cytb*. Ceci est la première description moléculaire de métacestodes d’origine animale de *T. multiceps* et de *T. hydatigena* en Mongolie intérieure, Chine. De nouvelles séquences nucléotidiques pourraient refléter des caractérisations génétiques endémiques des deux cestodes. Ces données sont utiles pour explorer la signification biologique et épidémiologique des variations intraspécifiques au sein des *Taenia*.

## Introduction

*Taenia* is a complicated genus of tapeworms composed of more than 40 accepted species, and in some of the species, subspecies have also been described [10,12]. Some members of the genus are responsible for taeniasis and/or cysticercosis in humans, and these helminthiasis diseases belong to the group of neglected tropical diseases. Coenurosis and abdominal cysticercosis are the most prevalent parasitic diseases worldwide in livestock; however, they primarily occur in sheep and goats in well-developed countries in areas of livestock husbandry [18,23]. The livestock are usually infected by ingesting grass contaminated with the eggs of *Taenia multiceps* (*T. multiceps*) and *Taenia hydatigena* (*T. hydatigena*) in the feces of definitive hosts, mainly including dogs and wolves [3]. These parasitic infections can cause a variety of clinical symptoms due to differences in the localization of cysts. *Coenurus cerebralis* is the most commonly found in the brain and spinal cord of sheep and goats, causing neurological disorders including ataxia, hypermetria, head deviation, headache, stumbling and paralysis [3]. The larva is also found in muscle, subcutaneous, and connective tissues, and other unusual locations [17,27]. Mature *cysticercus tenuicollis* cysts are usually found in the omentum, mesentery and peritoneum, less frequently in the pleura and pericardium and occasionally in the lungs, kidneys, brains, ovaries, uterine tubes, uterus, cervix, and vagina, while migrating larvae can be found mostly in the liver parenchyma [24,26]. In heavy infections, these larvae cause hemorrhagic and fibrotic lesions of liver parenchyma known as “hepatitis cysticercosa” [24,26]. Coenurosis and abdominal cysticercosis are usually fatal to livestock and cause huge losses to the economy due to condemnation of infected muscle and offal [9,21]. Human cases of coenurosis mostly result from accidental infections, and this disease has been found in South Africa, Europe, India, the USA, Brazil and Israel [13]. However, to date, only one human case of abdominal cysticercosis was recorded, which came from a Chinese four-year-old boy suffering from a stomach ache [31].

In China, *coenurus* *cerebralis* and *cysticercus tenuicollis* have been detected in animals from more than 20 provinces, and in western and north-western areas, where livestock husbandry is relatively developed, the species are mainly endemic. Infection rates are 2.7% to 53.0% for *coenurus* *cerebralis* and 1.3% to 12.5% for *cysticercus tenuicollis* in sheep in some provinces, and no epidemiological data are available in goats for either of the two parasites, and their genetic characterization is rarely recorded [14]. Northern Inner Mongolia is one of the most well-developed areas of livestock husbandry in China, accounting for up to 30% of total Chinese livestock production. To date, there have been only a few reports of *T. multiceps* and *T. hydatigena* in animals in this area, and they were all conducted in the 1990s [7,30,33]. To date, no information is available on genetic characterization of *coenurus* *cerebralis* and *cysticercus tenuicollis* in goats and sheep.

Mitochondrial (mt) genes are widely used as genetic markers and are preferred to nuclear genes in analyzing genetic characterizations and assessing genetic relationships of *Taenia* cestodes because of the maternal inheritance, higher mutation rate, rapid evolutionary rate, lack of recombination, and conserved structure [15]. In the present study, we molecularly confirmed one sheep- and five goat-derived *coenurus cerebralis* cysts of *T. multiceps* and two goat-derived *cysticercus tenuicollis* cysts of *T. hydatigena,* and analyzed their genetic variations at three mt gene loci (*cox1*, *nad4*, and *cytb*) at the nucleotide and amino acid levels. The biological and epidemiological significance of intra-specific variations of metacestode cysts of *T. multiceps* and *T. hydatigena* was explored.

## Materials and Methods

### Source of metacestode cysts

Inner Mongolia is located in the north of China (53°23’N, 97°12’E), including most of China’s border with Mongolia and a small section of the border with Russia. It has a wide variety of regional climates due to its elongated shape. Most of this region is a plateau averaging around 1,200 meters in altitude. The annual average temperature is 6 °C, with temperatures below 0 °C reached in October in the north and November in the south. During the period of time from July to September 2014, *coenurus cerebralis* cysts of *T*. *multiceps* (five from goats and one from a sheep) and *cysticercus tenuicollis* cysts of *T. hydatigena* from two goats were collected from an abattoir in Inner Mongolia. Specimen information, including code, species, location and size of each cyst in diameter as well as host sources, is shown in Table 1.

**Table 1:** Specimen code, location and size as well as host sources of metacestode cysts.

Specimen code	*Taenia* species	Host	Location	Cyst diameter (cm)
IM1	*T. multiceps*	Sheep	Mesentery of small intestine	3.5
IM2	*T. multiceps*	Goat	Brain	1.5
IM3	*T. hydatigena*	Goat	Intestine	2.2
IM4	*T. hydatigena*	Goat	Intestine	1.3
IM5	*T. multiceps*	Goat	Brain	1.8
IM6	*T. multiceps*	Goat	Brain	1.4
IM7	*T. multiceps*	Goat	Brain	2.1
IM8	*T. multiceps*	Goat	Brain	1.6

### Processing of endocysts and DNA extraction

Cyst fluid of each of the larval cestodes was drained while the endocysts were preserved in 70% ethanol at 4 °C until further molecular analysis. Genomic DNA of larval cestodes was extracted from approximately 25 mg cyst wall positive for scoleces using the DNeasy blood and tissue kit (Qiagen, Germany), according to the manufacturer’s instructions. DNA was eluted in 200 µL of Buffer AE and was stored at −20 °C until further use in PCR analysis.

### PCR amplification

The partial cytochrome c oxidase subunit I (*cox1*), NADH dehydrogenase subunit 4 (*nad4*) and cytochrome b (*cytb*) genes of eight metacestode cysts of *T. multiceps* and *T. hydatigena* were amplified in the present study. All the primers used here and the cycle parameters of PCR amplifications were described previously, and the objective gene fragments with the size of 455 bp, 667 bp and 570 bp were acquired, respectively [5,8,32]. TaKaRa Taq DNA Polymerase (TaKaRa Bio Inc., Tokyo, Japan) was used for all the PCR amplifications. A negative control with no DNA was included in all the PCR tests. All the PCR products were subjected to electrophoresis in 1.5% agarose gel and visualized by staining the gel with ethidium bromide.

### DNA sequencing and data analysis

All the PCR amplicons of the excepted size were sequenced with PCR primers at each locus on an ABI PRISMTM 3730 DNA Analyzer (Applied Biosystems, Carlsbad, CA, USA), using a BigDye Terminator v3.1Cycle Sequencing kit (Applied Biosystems, USA). The accuracy of the sequencing data was confirmed by sequencing in both directions. Nucleotide sequences obtained in the present study were congregated manually and were then subjected to BLAST searches (http://www.ncbi.nlm.nih.gov/blast/). Aligned sequences were compared and analyzed with each other and reference sequences downloaded from GenBank using ClustalX 1.83 (http://www.clustal.org/).

### Phylogenetic analysis

To present the genetic relationship of *T. multiceps* and *T. hydatigena* isolates obtained in the present study and published in GenBank as well as other members of the genus *Taenia*, the phylogenetic trees of the *cox1*, *nad4* and *cytb* gene sequences were constructed, respectively, using the neighbor-joining method with a Kimura-2-parameter model. The reliability of these trees was assessed using the bootstrap analysis with 1000 replicates.

## Results and Discussion

**Figure 1:**
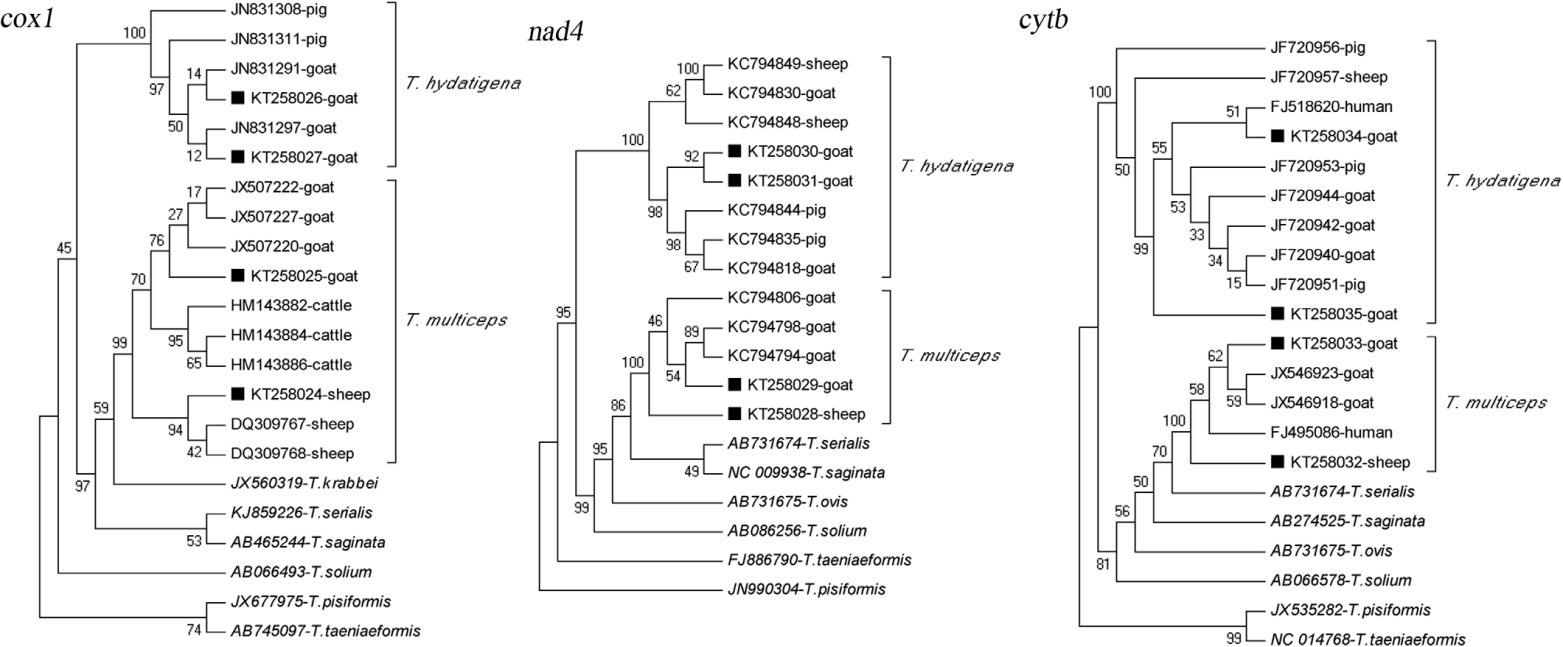
Phylogenetic relationship of *T. multiceps* and *T. hydatigena* isolates obtained in the present study and other *T. multiceps* and *T. hydatigena* isolates as well as other members of the genus *Taenia* as inferred by neighboring-joining analysis of the *cox1*, *nad4* and *cytb* gene sequences based on genetic distances calculated by the Kimura 2-parameter model. The reliability of these trees was assessed using a bootstrap analysis with 1,000 replicates. The GenBank accession number and the host of origin are given for each isolate of *T. hydatigena* or *T. multiceps*. Black squares indicate the sequences of *T. hydatigena* or *T. multiceps* from the present study.

In the present study, eight metacestode cysts were successfully amplified at the *cox1*, *nad4* and *cytb* loci. The results of pairwise comparison of nucleotide sequences indicated the genetic heterogeneity within coenurus cerebralis and cysticercus tenuicollis at three mt loci. Two haplotypes were obtained at each locus for both of them. Meanwhile, a lesser degree of intra-specific nucleotide variation was observed within *coenurus cerebralis* in the mt genes of *cox1 (*1.18%), *nad4* (0.61%) and *cytb* (0.52%). Only two base mutations caused amino acid changes, which occurred at the *nad4* locus. *Coenurus cerebralis* was observed to have more common genetic variations at the *cox1* locus in the present study than in two recent studies, where five and seven haplotypes were defined among 105 and 102 *coenurus* *cerebralis* isolates from Greece and Iran, respectively [2,22]. For *cysticercus* *tenuicollis*, the degrees of intra-specific nucleotide variation were 0.24%, 0.46% and 0.35% at the *cox1*, *nad4* and *cytb* loci, respectively. Only three base mutations caused amino acid changes, with one and two in the *nad4* and *cytb* genes, respectively. Homology analysis of nucleotide sequences revealed that ten of twelve representative nucleotide sequences were not described previously (Table 2). Representative nucleotide sequences obtained in this study were deposited in the GenBank database under the accession numbers KT258024 to KT258035. These novel sequences might reflect endemic genetic characterizations of *T. multiceps* and *T. hydatigena*. In phylogenetic analysis, it was observed that at the *cox1* locus, there was a lower bootstrap value (below 50) between *T. hydatigena* and *T. multiceps* as well as *T. krabbei*, *T. serialis* and *T. saginata* (Figure 1). The result might be related to the partial *cox1* gene sequences analyzed. Meanwhile, it also implied the amplified gene fragment (423 bp at nucleotides from 719 to1142) was not good enough in resolution for inter- and intra-variation of *Taenia* species. Sequence analysis of the complete *cox1* gene will be performed in the future to provide accurate phylogenetic relationships of the members of the genus *Taenia* as well as genetic variation within *Taenia* species.

**Table 2:** Homology analysis of *T. multiceps* and *T. hydatigena* isolates at the three mitochondrial loci.

*Taenia* species	Loci amplified	Accession no.^a^ (Specimen code)	Accession no.^b^	Homology	Codon^c^/Amino acid (Nucleotide Position)^d^
*T. multiceps*	*cox1*	KT258024 (IM1)	EF393620	100%	
		KT258025 (IM2, IM5-8)	JX507230	99.53%	CC(A to G)/P(76); GT(T to C)/V(310)
	*nad4*	KT258028 (IM1)	KC794808	99.09%	(G to A)TT/(V to I)(88); GC(C to T)/A(225); (G to A)TG/(V to M)(529); AC(T to C)/T(570); AG(A to G)/S(645); TC(G to A)/S(654)
		KT258029 (IM2, IM5-8)	KC794808	99.70%	GC(C to T)/A (225); TC(G to A)/S(654)
	*cytb*	KT258032 (IM1)	FJ495086	100%	
		KT258033 (IM2, IM5-8)	JX546908	99.83%	TC(A to G)/S(379)
*T. hydatigena*	*cox1*	KT258026 (IM3)	AB792724	99.53%	TA(C to T)/Y(13); TG(G to A)/W(397)
		KT258027 (IM4)	AB792724	99.76%	TA(C to T)/Y(13)
	*nad4*	KT258030 (IM3)	KC794844	98.94%	TT(A to G)/L(39); AT(C to T)/I(69); TA(C to T)/Y(150); G(A to G)G/(E to G)(323); TT(A to G)/L(615); TG(C to T)/C(651); TC(G to A)/S(654)
		KT258031 (IM4)	KC794845	98.94%	TT(A to G)/L(39); AT(C to T)/I(69); TA(C to T)/Y(150); CT(A to G)/L(159); TG(G to A)/W(333); TG(C to T)/C(651); TC(G to A)/S(654)
	*cytb*	KT258034 (IM3)	FJ518620	99.83%	AT(A to G)/(I to M)(22)
		KT258035 (IM4)	FJ518620	99.83%	GG(C to T)/G(46)

a Accession no. indicating the representative sequences obtained in the present study.

b Accession no. indicating that the reference sequences here have the largest similarity with those obtained in the present study.

c All the base changes in brackets are from reference sequences to representative sequences obtained in the present study.

d Nucleotide position numbers are according to the sequences obtained in the present study, with the beginning of the coding region of each mt gene as position no. 1.

Currently, the significance of the nucleotide and amino acid changes within *coenurus cerebralis* or *cysticercus tenuicollis* is unclear. However, genetic variability was reported previously to lead to phenotypic differences in many cestodes [16]. Varcasia *et al.* also pointed out that the presence of some clinical variations in coenurosis can indicate the existence of genetic intra-specific variability within *T. multiceps* [29]. It was speculated that the intra-specific sequence variations might represent the ability of these parasites to rapidly adapt to new intermediate host species as well as sites of infection occupied by the larval stages in different hosts [27]. In our study, five *coenurus cerebralis* cysts from the brain produced the same nucleotide sequence and were different from those from the mesentery of the small intestine at each locus (*cox1*, *nad4* and *cytb*). A previous comparative study of experimental cerebral and non-cerebral coenurosis in goats indicated that the cysts of two groups were 100% identical to each other at the *cox1* and *nad1* loci [1]. In a recent study conducted in Turkey, different *cox1* haplotypes of *coenurus cerebralis* were found in the same parasitic body part (brain) in sheep [25]. Identical sequences of *coenurus cerebralis* were also found in different hosts and different body parts: *cox1* sequences (396bp) in the muscle of a goat (KT253993) and in the brain of a sheep (KT253934) [19]. In an investigation of the genetic variation and population structure of *cysticercus tenuicollis* from intermediate hosts, *cysticercus tenuicollis* isolates from sheep were observed to be differentiated from those of goat and pig origin at the *nad1* locus; in addition, the goat-derived isolates were genetically different from adult *T. hydatigena* as indicated by the statistically significant Fst value [4]. Whether sequence differences are related to clinical features and/or organ preference needs to be explored in the future by analyzing other *coenurus cerebralis* and *cysticercus tenuicollis* cysts.

Variability in measurements of *coenurus cerebralis* cyst size has been documented. In the present study, the average size in diameter was 1.98 cm for *coenurus cerebralis* cysts and 1.75 cm for *cysticercus tenuicollis* cysts. *Coenurus cerebralis* cysts are considered to be typically 2–5 cm in diameter, and occasionally exceed 10 cm [13]. In a necroscopy, two cysts (49 and 23 cm^3^) were found in the brain of a bull in Italy [28]. In the United Arab Emirates, the size of the *coenurus cerebralis* cysts varied between one and 40 cm^3^ in goats; meanwhile, the shape of the cysts was variable according to their location: elongated when located in the middle of muscular fibers or globular if recovered in the abdominal cavities or under the skin [27]. For *T. hydatigena*, a cyst attached to the greater omentum was measured 15×15×5 cm in a rhesus macaque [11]. According to a necropsy report for a goat, two small-sized cysticerci vesicles (around 2 cm in diameter) were observed in the peritoneal cavity of a 3-year-old animal in Portugal, while the other vesicle (6 cm in diameter) was found inside fetal membranes [20]. So far, no reasonable explanations are given about why cysts appear markedly different in size despite the living time of cysts in hosts. This might also be related to the affected organs.

## Conclusion

The present study described molecular characterization within *T. multiceps* and *T. hydatigena* in Inner Mongolia, China for the first time. The findings of different haplotypes at the three mt loci suggest common genetic variations of *coenurus cerebralis* and *cysticercus tenuicollis* in goats/sheep in the investigated area. These data will provide baseline information for future studies on the molecular epidemiology of *T. multiceps* and *T. hydatigena* as well as on the biological and epidemiological significance of intra-specific variations within both *Taenia* cestodes. However, in the present study, six *coenurus cerebralis* cysts were analyzed and five of them were collected from brains of goats, while the remaining one was collected from the small intestine mesentery of a sheep, which is different from a previous report that cerebral coenurosis occurred mostly in sheep and non-cerebral coenurosis mostly in goats [6]. This might be related to the small number of *coenurus cerebralis* cysts collected in the present study. In spite of this, based on the fact that dogs play an important role in the maintenance and the transmission of *T. multiceps* and *T. hydatigena* to animals and occasionally to humans, future work will focus on the epidemiological study of taeniasis in dogs, as well as evaluation of economic losses caused by *T. multiceps* and *T. hydatigena*. These data will also be valuable to develop suitable control strategies to avoid cestode infections in animals and humans.

## Competing interests

The authors declare that they have no competing interests.
